# Dual acquisition scheme–based optical coherence tomography 3D angiography

**DOI:** 10.1117/1.JBO.30.5.056004

**Published:** 2025-05-08

**Authors:** Junxiong Zhou, Wei Chen, Jianbo Tang

**Affiliations:** Southern University of Science and Technology, Guangdong Provincial Key Laboratory of Advanced Biomaterials, Department of Biomedical Engineering, Shenzhen, China

**Keywords:** optical coherence tomography angiography, cerebral vasculature, dynamic imaging

## Abstract

**Significance:**

Optical coherence tomography angiography (OCTA) is a noninvasive technique dedicated to high-resolution microvasculature imaging. However, the projection artifacts of large pial vessels make it difficult to visualize the underlying microvessels, challenging its 3D vascular imaging ability.

**Aim:**

We propose a dual acquisition scheme–based 3D OCTA method aimed at simultaneously mitigating projection artifacts and enhancing the detection of capillary networks.

**Approach:**

In this study, we introduce an approach incorporating a dual data acquisition scheme with optimally oriented flux (OOF) filtering to address this problem. The repeated A-scan acquisition scheme and corresponding data processing algorithm were used to address the projection artifact issue underneath large pial vessels, whereas repeated B-scan acquisition-based data processing was used to image the capillary network.

**Results:**

With such a processing scheme, the projection artifacts can be effectively suppressed, whereas the high detection sensitivity to small vessels of repeat B-scan OCTA can be preserved, thus enabling high-sensitivity 3D imaging of the cerebral vasculature after OOF filtering.

**Conclusions:**

The results demonstrate the capability of the proposed method for 3D OCTA imaging, which may play an important role in cerebral microvascular dysfunction-related disease studies.

## 1 Introduction

The changes in microvascular structure play an important role in the pathogenesis of neurovascular diseases.^1^ For instance, capillary dysfunction significantly reduces the efficiency of oxygen extraction in tissues, constituting a major factor in brain function degeneration.^2^ This has driven the increasing need for three-dimensional imaging of microvascular networks. Optical coherence tomography angiography (OCTA) is a powerful technique for imaging the microvascular networks, enabling quantitative assessment of the evolution of blood vessel structure.^3^ The OCTA imaging of blood vessels relies on detecting dynamic contrast. Red blood cells (RBCs) move quickly within the vessel network, which leads to signal fluctuation, whereas the detected signal from stationary tissue is relatively stable. Thus, by calculating the difference of OCT signals for the same location but at different time points, OCTA can image the blood vessel structure based on the dynamic contrast from the moving RBCs.

However, the issue of projection artifacts beneath large blood vessels presents a challenge for the three-dimensional imaging of the vascular network.^4^^–^^9^ The projection artifacts arise from the extended dynamic signal of the photons, which may have experienced multiple scattering events with moving RBCs.^10^ These multiple scattered photons carry dynamic information and have a light path longer than that of the directly reflected photons. As a result, the dynamic signal from such photons may extend into regions beneath the blood vessel when using OCT to obtain their “depth” (the photons’ travel length), creating false blood flow signals, i.e., the projection artifacts.^11^ To address the blood vessel projection artifacts, several strategies have been proposed. For instance, Li et al.^12^ combined enhanced OCTA images by merging large masks and microvessels with top-hat enhancement and optimally oriented flux (OOF) algorithms, effectively segmenting microvascular networks. Meanwhile, Zhang et al.^13^ have approached the issue by eliminating the projection artifact using a lower decorrelation value in each axial scan line. In addition, Stefan and Lee^14^ designed neural network models to suppress the projection artifacts. Nonetheless, the application of deep learning methods from pretrained models to new datasets often requires manual correction by professional technicians to remove artifacts while preserving the fine details of capillary structures in the 3D vasculature. Therefore, there is a pressing need for a direct and efficient method to accurately reconstruct the 3D cerebral microvascular network.

In our previous work, we introduced the adaptive decorrelation analysis ($Ag_{1}$-OCTA) method as a solution for achieving high-quality 3D vascular imaging using OCT.^15^ Although this approach effectively addresses the issue of projection artifacts, it presents a drawback in terms of reduced capillary detection. The $Ag_{1}$-OCTA utilizes an M-mode scan protocol (repeated A-scan) to assess voxel dynamic contrast, which is limited to a brief observation time of ∼0.66  ms. Consequently, the decorrelation signal of capillaries is relatively minor compared with swiftly flowing large vessels, leading to diminished detection of slower microcirculatory flows. By contrast, conventional OCTA technique^16^ calculates the dynamic contrast from consecutive B-scans, which provides a longer observation time/interval of ∼10  ms. This extended observation time between two adjacent B-scans can generate more dynamic variations, thereby leading to stronger projection artifacts but significantly enhancing the detection of capillary networks.

In this study, we propose a dual acquisition scheme–based 3D OCTA method aimed at simultaneously mitigating projection artifacts and enhancing the detection of capillary networks. Briefly, two sets of OCT data were acquired at the same region using repeated B-scan acquisition and repeated A-scan acquisition, respectively. The large vessel projection regions were identified, and then, a short observation and decorrelation calculation time were employed to suppress the projection artifacts of large vessels from the repeat A-scan dataset. Meanwhile, the blood flow information outside of the large vessel projection regions were obtained from the repeat B-scan dataset, which provides high detectivity of slow-flowing small vessels. Finally, use OOF filters to enhance the connectivity of capillaries. We compared the proposed method with regular OCTA and $Ag_{1}$-OCTA by imaging microvascular networks at different depths and further compared it with other image enhancement approaches. The results demonstrated the ability of the proposed method to suppress artifacts and enhance dynamic signals in capillaries.

## 2 Materials and Methods

### 2.1 Experimental Setup and Scanning Protocol

**Fig. 1:**
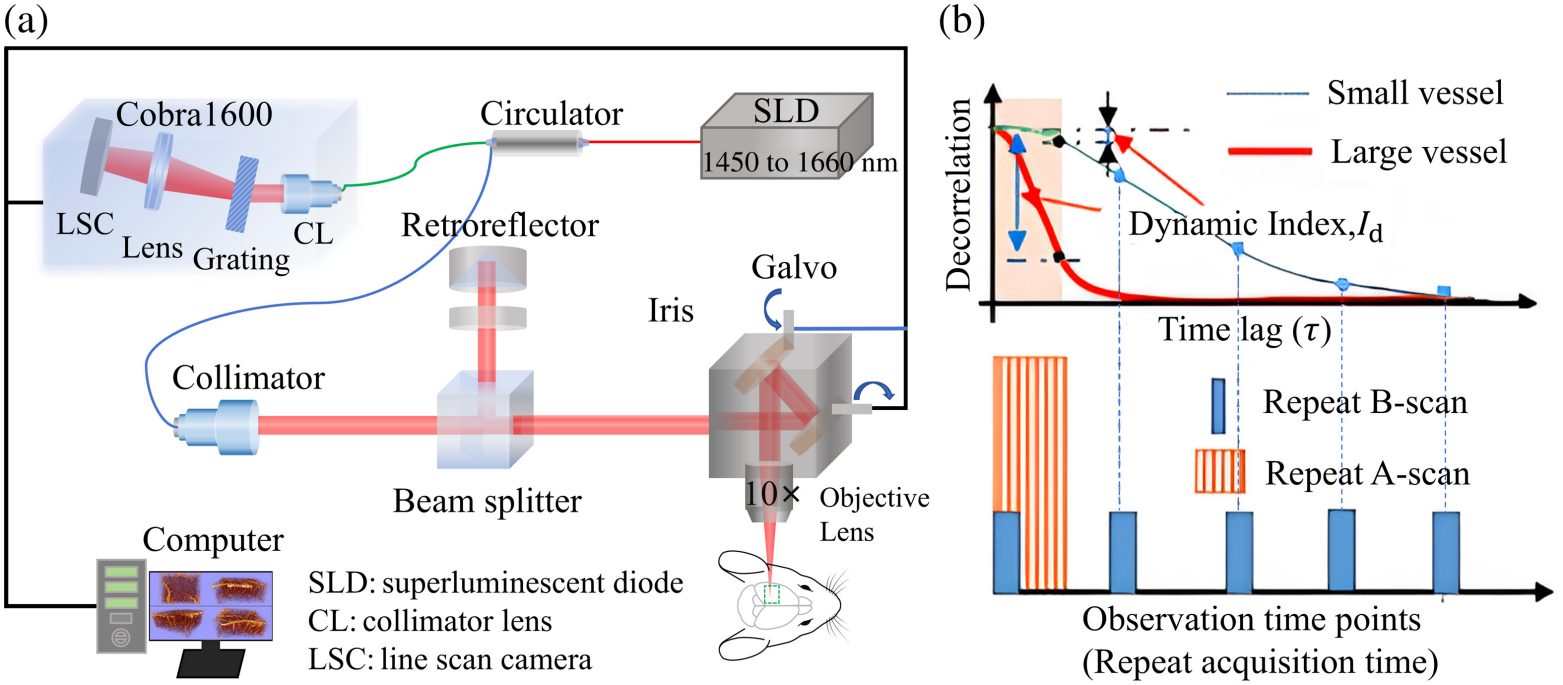
OCT setup and data acquisition strategies. (a) The custom-built OCT experimental setup. (b) For each location, repeated A-scan acquires more data but much shorter observation time, whereas repeated B-scan acquires less data (five repeats) but at a longer time span. For the short observation time, the signal decorrelation is small for small vessels, leading to weak detectability.

Figure 1(a) shows the custom-built OCT setup incorporating a dual-superluminescent diode broadband light source with a center wavelength of 1550 nm and a bandwidth of 210 nm, resulting in an axial resolution of 3.7  μm in the brain. A 10× objective was used to achieve a lateral resolution of 3.5  μm. The region of interest (ROI) was $1.8\times 1.8\;{\mathrm{mm}}^{2}$ with 600×600 transverse positions. In this study, the data were acquired with repeated B-scan and repeated A-scan protocols. For repeated B-scan acquisition, five repeated B-scans were acquired sequentially at each transverse position before moving to the next frame, and the frame interval was ∼10  ms. These repeated B-scans were collected along the same x-direction to ensure repeat accuracy for the detection of the same location. After completing the B-scan acquisition, repeated A-scans (50 repeats) were performed for the same region at an A-line rate of 76 kHz. The total data acquisition time was ∼30  s for repeated B-scans and 6 min for repeated A-scans acquisitions, respectively. To capture the entirety of the cortical vasculature, we acquired six datasets across various layers, each with a focal depth difference of ∼180  μm. We further implemented a cosine similarity algorithm to discern the shared sections among adjacent volumes, enabling us to seamlessly stitch and amalgamate them into a cohesive 3D volumetric dataset. Although there are only five time point signals in repeated B-scans, its time delay is longer than M-mode at the same position [Fig. 1(b)].

Figure 1(b) compares the observation time (acquisition time) and the signal decorrelation between the repeated A-scan and repeated B-scan protocols.^17^ Generally, rapid blood flow in large vessels results in a faster decorrelation, whereas in smaller vessels, the flow is slower, and it takes a longer time to be decorrelated. As a result, the detection of slow flows is lower compared with that of large vessels for a short observation time (repeated A-scan acquisition), as indicated by the dynamic index ($I_{d}$) Fig. 1(b). By contrast, the equivalent observation time is significantly longer for the repeat B-scan acquisition, which often leads to a complete decorrelation for both large and small vessels (indicated by blue dots for small vessels). Although the extended observation time enhances the detection of blood flow, it also strengthens the dynamic index in regions beneath the large pial vessels, leading to the issue of blood flow projection artifacts. Therefore, enhancing the detection of small vessels and mitigating the projection artifacts are needed to realize high-quality 3D imaging of the blood vessel network.

### 2.2 Dual Acquisition Scheme–Based 3D OCT Angiography

OCTA images blood vessels from static tissue by detecting the dynamic contrast from the OCT signal, which was obtained at different time points for the same location. The repeated B-scan method is prone to generating projection artifacts beneath large pial vessels, whereas the repeated A-scan method, due to its short observation time at the same location, has limited sensitivity in detecting capillaries. To improve the detection of small vessels and suppress the projection artifacts simultaneously, we propose a dual acquisition scheme–based OCTA approach that combines the advantages of enhanced detection of small vessels with OCTA and the projection artifacts suppression ability of repeated A-scan analysis ($Ag_{1}$-OCTA).

**Fig. 2:**
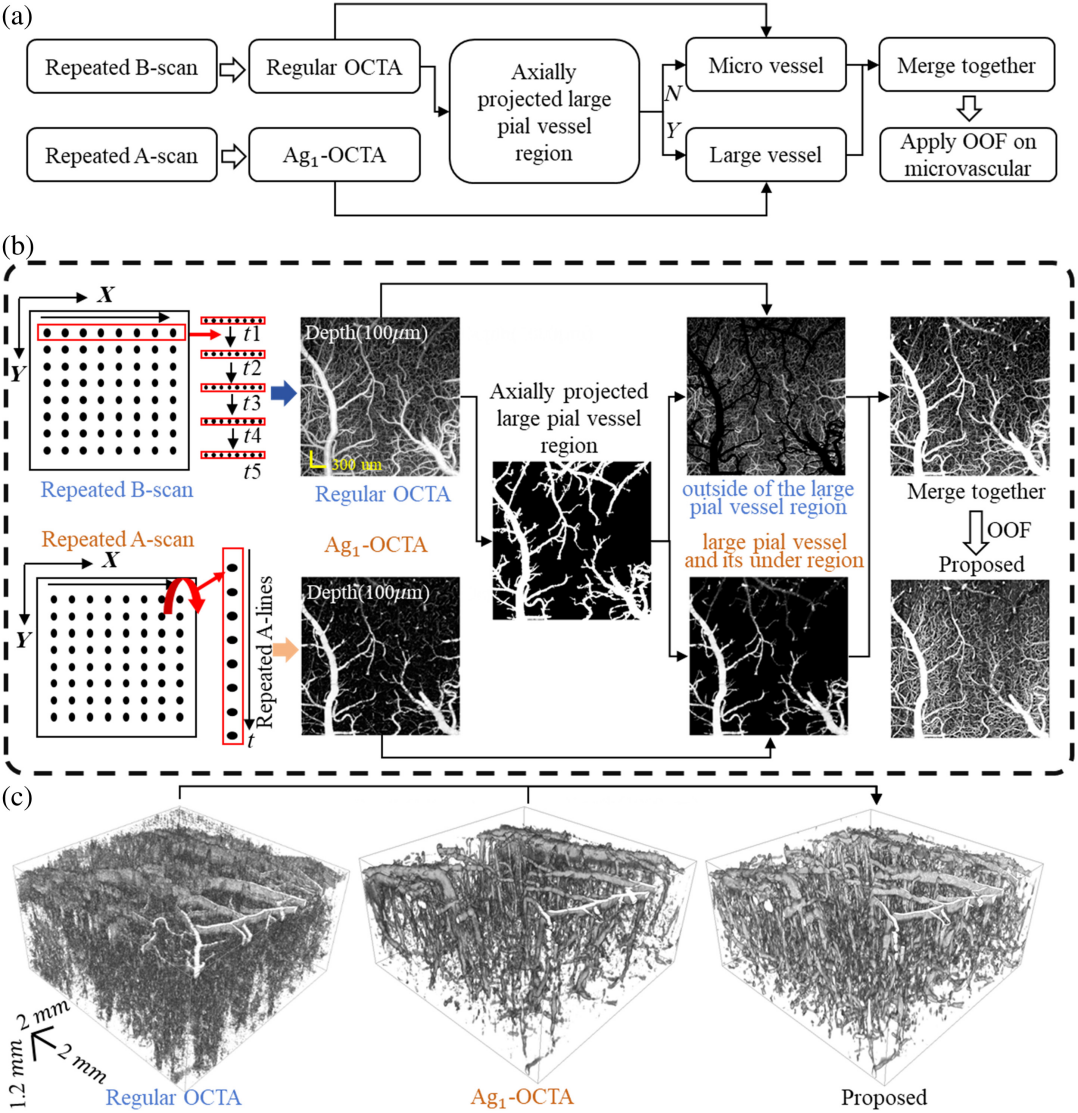
Data processing of the dual acquisition scheme–based OCTA. (a) and (b) Starting with five repeated B-mode acquisitions and M-mode acquisition with 50 repeated A-lines, we obtained microvasculature using regular OCTA and $Ag_{1}$-OCTA, and a large vessel mask was generated by diameter-based thresholding. Extract capillary regions and large pial vessels through the mask and then merge them together. Finally, apply OOF to the obtained results. (c) The comparison of 3D microvasculature rendering results among regular OCTA, $Ag_{1}$-OCTA, and the proposed method.

Figure 2 illustrates the detailed workflow of the proposed method. First, regular OCTA and $Ag_{1}$-OCTA algorithms were applied to obtain the microvasculature 3D images based on repeated B-scan and repeated A-scan acquisitions, respectively. Then, a maximum intensity projection along the axial direction (MIP) image of the blood vessels was obtained based on the OCTA result. Based on the MIP image, a binary mask of large vessels was generated using a diameter-based thresholding method (threshold set at 20  μm). This mask was then projected along the axial direction to create a 3D mask of large vessel regions. Within the large vessel region, the $Ag_{1}$-OCTA method was applied to suppress projection artifacts, whereas capillaries outside these regions were obtained using regular OCTA data processing. Finally, the brightness levels of the two subvolumes were adjusted to ensure uniform visualization, and the OOF method was applied to enhance microvascular structures and improve the visibility of fine capillaries. The proposed method integrates the advantages of repeated B-scan OCTA for detecting small vessels and repeated A-scan $Ag_{1}$-OCTA for suppressing projection artifacts beneath large pial vessels. Figure 2(c) shows the 3D microvascular rendering results obtained from repeated B-scans (regular OCTA), repeated A-scans($Ag_{1}$-OCTA), and the proposed method.

#### 2.2.1 Repeated B-scan with longer observation time enhances the detection of slow-flowing capillaries

A prominent example of regular OCTA technology based on complex signals in optical microvascular imaging is optical microvascular angiography (OMAG), first proposed by Wang et al..^18^ The dynamic contrast of blood flow signals is obtained by calculating the average of the absolute values of the response difference among consecutive B-scan paired complex signals.

$$I_{d}=\frac{1}{N-1}\overset{N-1}{\underset{t=1}{\sum }}|R(t+1)-R(t)|,$$ (1)

where N represents the number of repeated B scans, in our study, we set the number of B-scans to 5, as increasing the number of repeats improves the signal-to-noise ratio (SNR). OMAG is now the most widely used regular OCTA technique for 3D imaging, providing high-resolution vascular structures down to the capillary level, allowing for the precise delineation of microvascular structure and blood flow dynamics. This approach accounts for phase variations among adjacent B-scans and utilizes these differences to correct errors induced by physiological motion, thereby improving image quality. OMAG imaging reduces sensitivity to tissue heterogeneity and signal attenuation using amplitude information from OCT signals rather than phase information. As a result, it enables better imaging of vessels in deep and highly attenuated regions, with sufficient interscan time among adjacent B-scans to recover slow-flowing capillaries.

#### 2.2.2 Repeated A-scan with adaptive decorrelation analysis suppresses blood vessel projection artifacts

We extracted 50 continuous OCT field signal series [R(t),0<t≤50] at each point. First, we calculated each voxel’s autocorrelation function $g_{1}(\tau )$ from the

$$g_{1}(\tau )=\frac{{\left\langle R^{*}(t)R(t+\tau )\right\rangle }_{t}}{{\left\langle R^{*}(t)R(t)\right\rangle }_{t}}.$$ (2)

Next, we determined the dynamic contrast index $I_{d}$ by taking the maximum decorrelation of the $g_{1}(\tau )$ within the defined decorrelation time following the first-time lag:

$$I_{d(1:n_{\tau })}=|g_{1}(1)|-\min|g_{1}(\tau {)}_{\left(1:n_{\tau }\right)}|,$$ (3)

where R*(t) is the complex conjugate, τ is the time lag, and ⟨ ⟩ represents ensemble averaging, || denotes magnitude, and $n_{\tau }$ is the decorrelation time point. $Ag_{1}$-OCTA analyzes the differences in decorrelation rate and $g_{1}$ phase shift between the regions affected by artifacts and the vascular voxels.^15^ Although the artifacts beneath the large vessels exhibit high decorrelation, their chaotic phase changes are key for $Ag_{1}$-OCTA to identify them. By adaptively selecting the decorrelation time ($n_{\tau }$) based on the characteristics of the vascular voxels and artifact regions, shorter decorrelation periods can be used to suppress projection artifacts in tail regions, whereas longer times enhance blood flow dynamic contrast index in vascular voxels. In regular OCTA imaging, projection artifacts under large vessels can obscure microvascular signals and reduce image quality. $Ag_{1}$-OCTA is specifically designed to suppress these artifacts, making it essential for studying microvascular structure as it not only reduces artifacts but also improves the dynamic signal of microvessels.

### 2.3 Optimally Oriented Flux Filtering

The intricate three-dimensional microvascular networks and dense capillary beds present challenges for accurate vessel extraction. Poor vessel visibility and image quality make it difficult to process OCTA images with low SNR.^19^ To enhance vessel structure continuity, we employed an OOF filter.^20^ The OOF filter approach, based on the Hessian matrix, detects and amplifies curvilinear structures in images, particularly tubular structures such as vessels. It determines vascular curvatures by calculating the second-order gradient vector of image brightness within a spherical region centered on local voxels, measuring the direction and magnitude of local blood flow. The OOF filter generates a detection response when the spherical region’s perimeter intersects the edges of curvilinear structures. The OOF filter’s curved structure detection is particularly effective when two structures are close, yielding responses from adjacent structures at closer locations.

The Hessian matrix is a second-order derivative matrix that describes the local curvature surrounding a pixel. For a given pixel at position f(x,y,z), the Hessian matrix H(f) is defined as follows:

$$H(f)=\{\begin{array}{ccc} \frac{\partial^{2}f}{\partial x^{2}} & \frac{\partial^{2}f}{\partial xy} & \frac{\partial^{2}f}{\partial xz}\\ \frac{\partial^{2}f}{\partial yx} & \frac{\partial^{2}f}{\partial y^{2}} & \frac{\partial^{2}f}{\partial yz}\\ \frac{\partial^{2}f}{\partial zx} & \frac{\partial^{2}f}{\partial zy} & \frac{\partial^{2}f}{\partial z^{2}} \end{array}\}.$$ (4)

Here, each element in the Hessian matrix represents the process of calculating the second-order partial derivative along the three directions xyz, respectively. The critical step in the OOF filter process is determining the optimal orientation for each pixel point where the flux is maximized. This orientation is identified by the eigenvectors of the Hessian matrix, specifically the eigenvector associated with the largest eigenvalue. For each pixel (x,y,z), the OOF filter computes the Hessian matrix H(f) and its eigenvalues $\lambda_{1}$, $\lambda_{2}$, and $\lambda_{3}$ along with the corresponding eigenvectors “$V_{1}$,” “$V_{2}$,” and “$V_{3}$.” The eigenvector “$V_{1}$” associated with the largest eigenvalue “$\lambda_{1}$” is chosen as the direction of maximum flux. The final OOF response map P(f) can be calculated using the following equation:

$$P(f)=\max\{\underset{r}{\max}\{-\frac{1}{r^{2}}\lambda_{1}(f,r)\},0\}.$$ (5)

In this equation, r is the scale parameter used to determine the optimal flux response at different scales. The equation effectively calculates the maximum negative value of the eigenvalues (scaled by “$\frac{1}{r^{2}}$”) and limits it to 0 to ensure nonnegative responses, emphasizing the tubular structures while suppressing background noise. The OOF filter is particularly beneficial for vessel segmentation and enhancement tasks due to its ability to improve low intensities and weakly connected capillaries. It was found that the OOF response to large blood vessels decreases with a larger r, so we applied OOF only to capillaries.

### 2.4 Animal Preparation

We acquired *in vivo* data from adult C57BL/6 mice prepared with a 5 mm diameter chronic cranial optical window. During the imaging session, the mice were anesthetized with 1% to 2% isoflurane. Following intralipid administration (20% intralipid solution, ∼120  μg) via orbital injection, both repeated B-scan acquisition (five repeated B-scans) and M-mode acquisition (50 repeated A-scans) were performed to obtain two sets of data from the same region. The animal experiment protocol was approved and supervised by the Animal Ethics Committee at the Southern University of Science and Technology.

## 3 Results

**Fig. 3:**
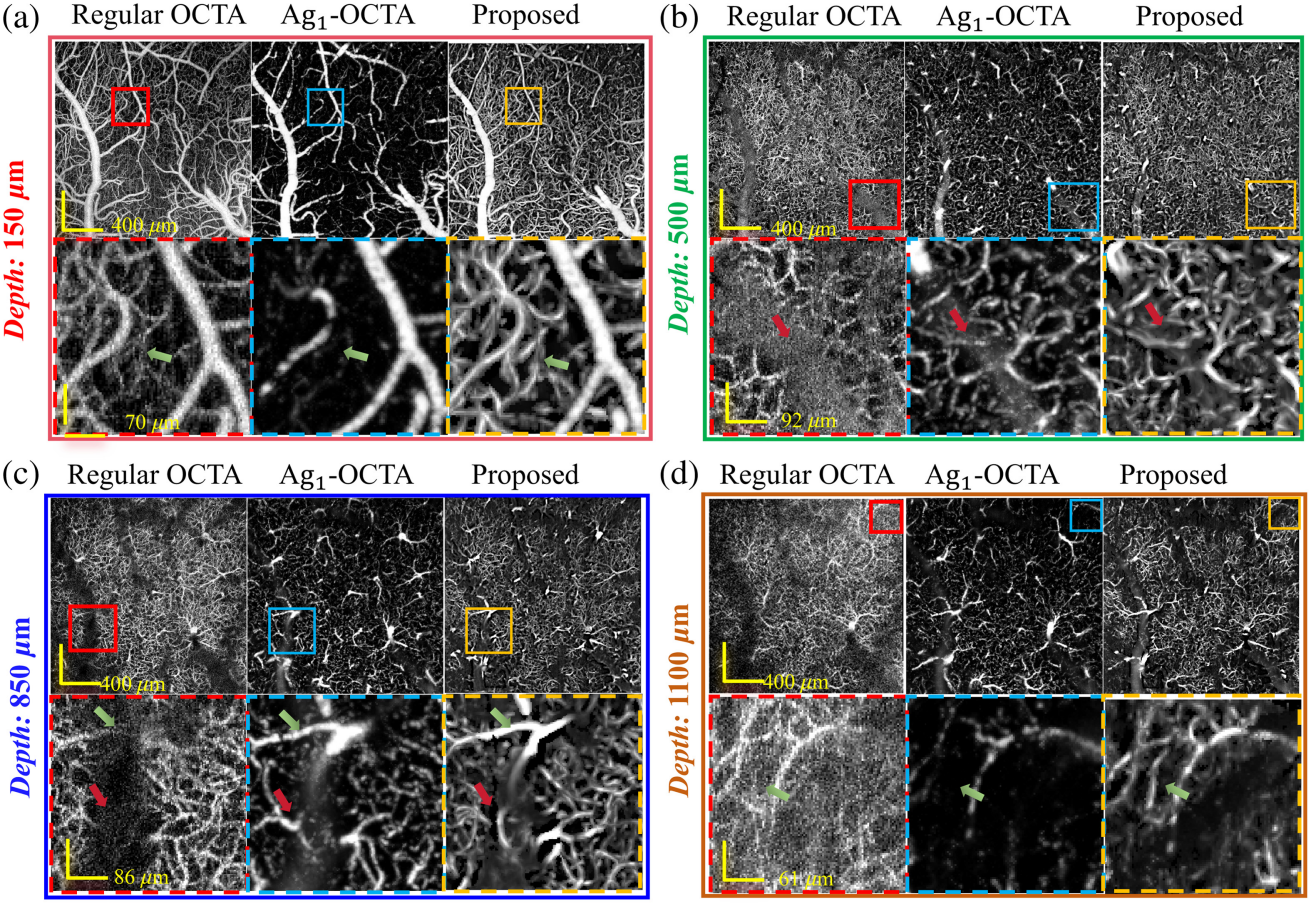
Comparison of the *en face* MIPs (with 100  μm stacks) results obtained by regular OCTA, $Ag_{1}$-OCTA and proposed method across various depths: (a) upper regions (150  μm), (b) middle regions (500  μm), and (c) and (d) and deeper tissue regions (850 and 1100  μm), respectively. Green arrows indicate the ability of the proposed method to enhance capillaries, whereas the red arrows indicate its suppressive effect on artifacts.

In Fig. 3, we first compared our method with the regular OCTA and previously proposed $Ag_{1}$-OCTA approaches for structure information. In the upper regions [100 to 200  μm, see Fig. 3(a)], both the regular OCTA and the proposed method show the dense capillary network, whereas the $Ag_{1}$-OCTA method obtains quite a few small vessels, which is due to the short observation time to obtain the dynamic information used by $Ag_{1}$-OCTA. In the middle regions [450 to 550  μm, see Fig. 3(b)], the regular OCTA method suffers from projection artifacts beneath the large vessels, where the dynamic contrast of some artifacts was even stronger than that of the small vessels. It was difficult to identify the microvascular morphology due to severe projection artifacts extending into deeper regions. By contrast, both $Ag_{1}$-OCTA and the proposed method can effectively suppress these projection artifacts.

We further evaluated the capillary imaging ability in deeper regions [800 to 900 and 1050 to 1150  μm, see Figs. 3(c) and 3(d)]. The number of incident photons decreases exponentially, particularly in areas beneath large vascular structures. Regular OCTA techniques, which use differences in light intensity to calculate the dynamic index, were severely affected by shadow artifacts beneath large vessels. By contrast, decorrelation-based $Ag_{1}$-OCTA and the proposed method effectively detected dynamic changes and obtained images with enhanced dynamic contrast. Again, the proposed method shows higher sensitivity to image the capillary network compared with the $Ag_{1}$-OCTA, as shown in the zoom-in images in Fig. 3(d).

**Fig. 4:**
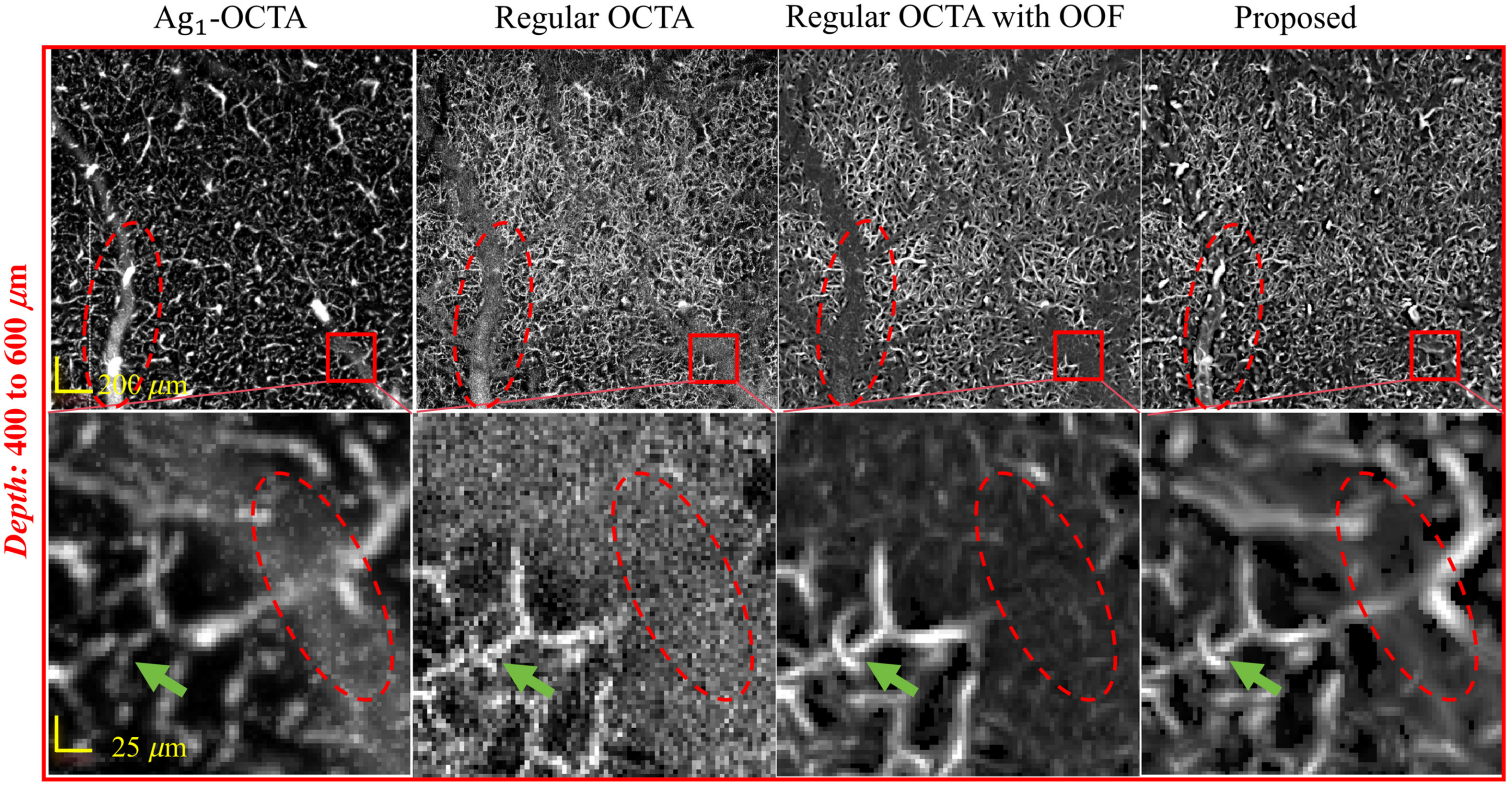
*En face* MIPs and zoomed-in images (using 200  μm stacks) obtained by $Ag_{1}$-OCTA, regular OCTA, regular OCTA with OOF, and proposed method at depth ranges of 400 to 600  μm. The green arrows highlight that the proposed method enables clear visualization of capillaries, whereas the red dashed box highlights the area affected by projection artifacts.

OOF is a vascular enhancement filter that processes image gradients at pixel boundaries to enhance vascular continuity and highlight intricate microvascular details. To evaluate the effect of OOF processing, we compared the results obtained with $Ag_{1}$-OCTA, regular OCTA, regular OCTA with OOF, and the proposed method, as shown in Fig. 4. As indicated by the green arrows, $Ag_{1}$-OCTA is challenged to capture the slow-flowing capillaries, whereas regular OCTA measured capillaries are affected by noise, making it difficult to distinguish vascular boundaries. By contrast, both regular OCTA with OOF and the proposed method produce clearer and higher SNR vascular images out of the large vessel region. In the tail artifact regions, as indicated by the red dashed box, regular OCTA suffers from pronounced trail artifacts beneath large pial vessels, obstructing the imaging of underneath vessels. Even after OOF processing, no blood flow signals are obtained in this region. $Ag_{1}$-OCTA can suppress tail artifacts, allowing the imaging of blood flow within the artifact region. Thus, by integrating blood flow signals detected via the two different mechanisms and applying OOF processing for capillary enhancement, the proposed method can successfully suppress artifacts beneath large vessels and enhance the dynamic contrast of capillary signals at the same time. This enables high-quality microvascular imaging, which is beneficial for quantitative capillary network analysis of the capillary network. Therefore, combining the dual-scheme data acquisition and OOF filtering can further enhance the vascular image quality.

**Fig. 5:**
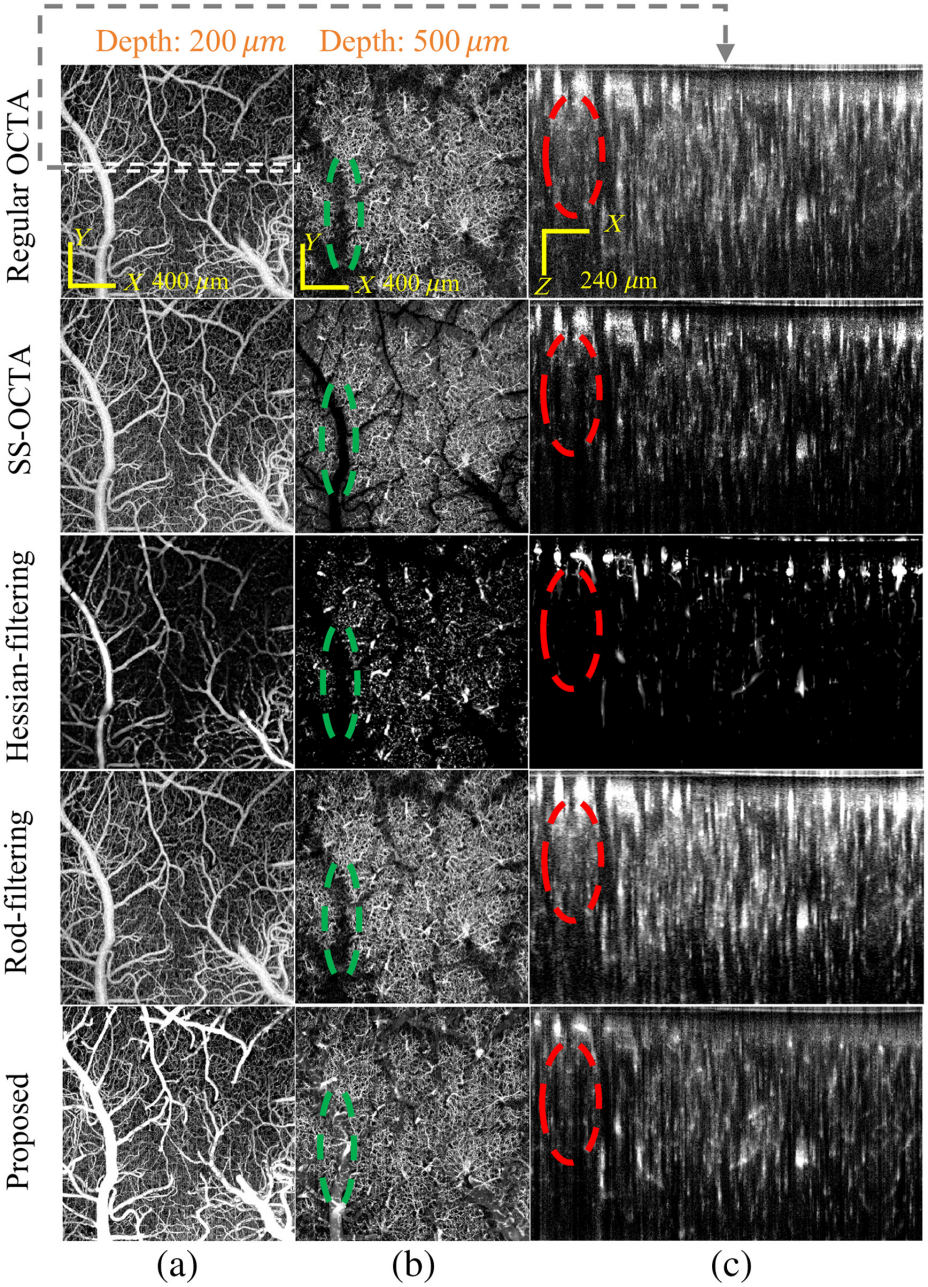
Comparison of the proposed method with other OCTA and postimage processing methods. Panels (a) and (b) show the *en face* MIP maps obtained at depth ranges of 200 to 350  μm and 500 to 650  μm. Panel (c) is the cross-sectional MIP slice marked by the white dashed box in the top-left figure.

In Fig. 5, we further compared the performance of the proposed method with the slab subtraction method (SS-OCTA)^21^ and two representative tubeness filtering methods, including the Hessian-based filtering^22^ and the rod filtering methods.^23^ At the XY cross-section at a depth of 200  μm, all methods, except the Hessian-based method, successfully image large vessels and dense capillary networks. The Hessian method, however, is constrained by the choice of the scaling factor, which results in a tradeoff to simultaneously preserve both large vessels and small capillaries, as shown in Fig. 5. In Fig. 5(c), the cross-sectional XZ slice shows that regular OCTA suffers from significant projection artifacts beneath large vessels, as indicated by the red dashed boxes. Although all other methods demonstrate the ability to suppress projection artifacts, the blood vessels underneath the large pial vessels cannot be detected by SS-OCTA, Hessian filtering, and rod filtering methods, as marked by green dashed boxes in Fig. 5. By contrast, taking advantage of tail artifacts suppression and enhanced small vessel detection, the proposed method can effectively image the vessels beneath large pial vessels, providing enhanced flow continuity.

## 4 Discussion and Conclusion

In this study, we employed a dual acquisition strategy using two different data processing algorithms. The first method employs the complex signal difference between two adjacent B-scans to calculate dynamic contrast. This approach utilizes a longer observation window (∼10  ms), which enhances the detection of capillaries. The second method uses an M-mode scan (repeat A-scan) to calculate the dynamic contrast of the voxel. This technique is particularly effective in resolving artifacts generated by tail shadows and in recovering weak signals from tiny capillaries. We further proposed to use the OOF filter on the obtained results, which helps to enhance the image quality. We were able to accurately reconstruct a 1.2×1.8×1.8 $(z\times x\times y)\;{\mathrm{mm}}^{3}$ microvascular network and demonstrate its efficacy in high-resolution, deep-penetration imaging of brain microvasculature.

The M-mode protocol offers a shorter interframe time interval, enabling more accurate phase changes, and can suppress the blood vessel projection artifacts with $Ag_{1}$-OCTA method. The physically relevant parameters affecting the performance of $Ag_{1}$-OCTA method are the time delays over which the autocorrelation function is calculated (i.e., autocorrelation time period, $n_{\tau }$) and the total observation time ($n_{t}$). A longer observation time can improve the detection of slowly moving signals but at the cost of longer data acquisition time and data size. Although as we demonstrated in our previous publication that the observation time can be reduced to 50,^17^ the $Ag_{1}$-OCTA is inferior to regular OCTA in detecting capillary networks as the signal variation/dynamic contrast is small in such a short observation time. Although the total acquisition time is longer using the M-mode data acquisition (6 min) compared with the regular OCTA data acquisition (∼30  s for 10 repeats/averaging), for each imaging location, the total acquisition time lapse is much shorter for the M-mode acquisition (∼0.66  ms) compared with the regular OCTA acquisition (∼50  ms, repeated B-scan), whereas the acquisition rate is much faster (0.013 ms versus 5 ms). Such a short acquisition interval enables $Ag_{1}$-OCTA to differentiate the tail artifacts from the vessel regions using $g_{1}$ analysis.

It is worth noting that averaging multiple volumes from regular OCTA images can help to enhance the SNR and improve the image quality, but it cannot address the blood vessel tail artifact issue.^24^ Therefore, by integrating the two acquisition schemes, the projection artifacts of large pial vessels can be suppressed using $Ag_{1}$-OCTA method, whereas the detection of small blood vessels is preserved with regular OCTA. Nonetheless, it is important to note that our research aims to achieve accurate three-dimensional vascular imaging. The M-mode data acquisition approach can be further extended for blood flow velocity measurements using dynamic light scattering optical coherence tomography (DLS-OCT).^25^ By integrating high-resolution cerebral microangiography with large-scale microvascular blood flow velocity measurements from DLS-OCT, our methodology enables a comprehensive analysis of the cerebral microvascular blood flow network down to the capillary level. This novel approach also allows for more precise quantification of the microvascular blood flow velocity distribution.

Regular OCTA is prone to bulk motion noise due to involuntary body movements such as breathing and heartbeat (see Fig. 5, regular OCTA). In our experiments, to reduce bulk motion, the mice were anesthetized with 1% to 2% isoflurane, and their skulls were stabilized using a custom headbar. In addition, we do five B-scan repetitions to reduce noise. Several postimaging processing methods have been proposed for artifact suppression in regular OCTA. For example, some approaches average the speckle variance of OCT B-scans across different spectral bands^26^ or subtract the average value of each A-scan.^27^ In this work, we tackle the long-standing challenge of OCTA blood vessel tail artifacts from the physical basis, whereas most of the previous methods were based on postimage processing. Our current work has three advantages: (1) effectively suppressing the blood tail artifacts, (2) preserving the flow of small vessels in the tail artifact background, and (3) enhancing the signal in both small and large vessel regions. In addition, the dual scheme–based image method introduced in this work is the first time that the suppression of blood vessel tail artifacts and the enhancement/preservation of small vessel signals have been achieved.

In conclusion, the proposed method outperforms the existing OCT-based vascular image enhancement techniques. The commonly used SS-OCTA can mitigate the tail artifact issue but suffers from the loss of vessel segments beneath the large pial vessels, resulting in disconnected flows. The Hessian-based method can enhance the flow signal but is limited by the tradeoff of filtering large vessels and small capillaries simultaneously. The rod filtering–based processing method tends to create regular circular structures, which is typically a consequence of excessive smoothing applied to the image, leading to the loss of vessel features. In addition, unlike the approach by Leahy et al.,^28^ which uses high NA confocal alignment to address the tail artifact issue and stack tiles at different focus to form a 3D vascular image, the proposed method addresses this issue from a physical perspective by integrating blood flow signals from different detection mechanisms. The high A-line rate but short observation time approach (repeated A-scan) allows for suppressing projection artifacts beneath large blood vessels, and the relatively slower rate but longer observation time approach (repeated B-scan) ensures the detection of capillaries. The combination of the dual-scheme advantages, along with the OOF filtering, greatly suppressed the tail artifacts and enhanced the vascular signal and its continuity.

It is worth mentioning that the extended data acquisition time for the proposed method compared with regular OCTA makes it not favorable for scenarios such as imaging the retina vasculature. However, it is useful to image the 3D microvasculature of the animal brain, which is important for studying brain function and diseases in the early stages. Such capabilities could offer reliable quantitative indicators for dynamic changes in capillary blood flow associated with brain functional disorders, including cerebral ischemia, stroke, and neural degeneration. Therefore, the proposed method would be a valuable tool in cerebral microcirculation-related neuroscience and brain disease research.

## Data Availability

Code and data underlying the results presented in this paper are not publicly available at this time but may be obtained from the authors upon reasonable request.
